# Mer-tyrosine kinase: a novel susceptibility gene for SLE related end-stage renal disease

**DOI:** 10.1136/lupus-2022-000752

**Published:** 2022-11-04

**Authors:** Sule Yavuz, Pascal Pucholt, Johanna K Sandling, Matteo Bianchi, Dag Leonard, Karin Bolin, Juliana Imgenberg-Kreuz, Maija-Leena Eloranta, Sergey V Kozyrev, Cristina M Lanata, Andreas Jönsen, Anders A Bengtsson, Christopher Sjöwall, Elisabet Svenungsson, Iva Gunnarsson, Solbritt Rantapää-Dahlqvist, ImmunoArray Development Consortium, DISSECT Consortium, Joanne Nititham, Lindsey A Criswell, Kerstin Lindblad-Toh, Lars Rönnblom

**Affiliations:** 1 Department of Medical Sciences and Science for Life Laboratory, Uppsala University, Uppsala, Sweden; 2 Science for Life Laboratory, Department of Medical Biochemistry and Microbiology, Uppsala University, Uppsala, Sweden; 3 National Human Genome Research Institute, National Institutes of Health, Bethesda, Maryland, USA; 4 Department of Clinical Sciences Lund, Rheumatology, Lund University, Lund, Sweden; 5 Rheumatology, Skåne University Hospital Lund, Lund, Sweden; 6 Department of Biomedical and Clinical Sciences, Linköping University, Linköping, Östergötland, Sweden; 7 Department of Medicine Solna, Karolinska Institute, Stockholm, Sweden; 8 Department of Rheumatology, Karolinska University Hospital, Stockholm, Sweden; 9 Department of Public health and Clinical Medicine, Umeå Universitet, Umeå, Sweden; 10 Broad Institute, Cambridge, Massachusetts, USA

**Keywords:** Lupus Erythematosus, Systemic, Polymorphism, Genetic, Autoimmune Diseases

## Abstract

**Objective:**

Lupus nephritis (LN) is a common and severe manifestation of SLE. The genetic risk for nephritis and progression to end-stage renal disease (ESRD) in patients with LN remains unclear. Herein, we aimed to identify novel genetic associations with LN, focusing on subphenotypes and ESRD.

**Methods:**

We analysed genomic data on 958 patients with SLE (discovery cohort: LN=338) with targeted sequencing data from 1832 immunological pathway genes. We used an independent multiethnic cohort comprising 1226 patients with SLE (LN=603) as a replication dataset. Detailed functional annotation and functional epigenomic enrichment analyses were applied to predict functional effects of the candidate variants.

**Results:**

A genetic variant (rs56097910) within the *MERTK* gene was associated with ESRD in both cohorts, meta-analysis OR=5.4 (2.8 to 10.6); p=1.0×10^-6^. We observed decreased methylation levels in peripheral blood cells from SLE patients with ESRD, compared with patients without renal SLE (p=2.7×10^-4^), at one CpG site (cg16333401) in close vicinity to the transcription start site of *MERTK* and located in a DNAse hypersensitivity region in T and B cells. Rs56097910 is linked to altered *MERTK* expression in kidney tissue in public eQTL databases. Two loci were replicated for association with proliferative LN: *PRDM1* (rs6924535, p_meta_=1.6×10^-5^, OR=0.58) and *APOA1BP* (*NAXE*) (rs942960, p_meta_=1.2×10^-5^, OR=2.64).

**Conclusion:**

We identified a novel genetic risk locus, *MERTK*, associated with SLE-ESRD using the data from two large SLE cohorts. Through DNA methylation analysis and functional annotation, we showed that the risk could be mediated through regulation of gene expression. Our results suggest that variants in the *MERTK* gene are important for the risk of developing SLE-ESRD and suggest a role for *PRDM1* and *APOA1BP* in proliferative LN.

**WHAT IS ALREADY KNOWN ON THIS TOPIC**

- Lupus nephritis (LN) is one of the most common major organ involvements in SLE that may progress to end-stage renal disease (ESRD) in approximately 10% of patients with LN.
- It is still unclear who will develop LN and who will progress to ESRD. Identification of genetic risk factors may lead to better risk assessment.

**WHAT THIS STUDY ADDS**

- Using two large SLE cohorts, a novel genetic locus, *MERTK,* was identified as associated with SLE-ESRD and replicated across different ethnicities.
- Functional potential of this gene and the immune cell types that are involved in mediating genetic risk in SLE-ESRD are highlighted using in silico tools.

**HOW THIS STUDY MIGHT AFFECT RESEARCH, PRACTICE OR POLICY**

- The ability to predict progression to ESRD may subsequently lead to therapeutic targets to prevent it.
- The results of this study support *MERTK* as a promising target for preventing ESRD in patients with LN.

## Introduction

Lupus nephritis (LN) affects up to 50% of patients with SLE and is potentially the most damaging manifestation.^1 2^ Although advances have been made through immunological discoveries and genetic association studies in SLE, the outlook for patients with LN has not been improved dramatically over the years, as around 10% still progress to end-stage renal disease (ESRD).^1 3^ Exact pathogenic mechanisms have yet to be fully elucidated. Mostly, immune complex-mediated inflammation initiates renal damage by different mechanisms; aberrant tissue repair and fibrosis, as a result of ongoing inflammation; cellular stress and hypoxia, contribute to the process leading to ESRD.^4^ Diffuse proliferative LN exerted the highest risk of ESRD in patients with LN development in a meta-analysis.^5^

The ethnic disparity in SLE and LN, with the highest disease burden in non-Caucasians,^6 7^ and familial clustering of ESRD in patients of African ancestry with SLE supports a genetic component to LN susceptibility and severity. Analyses of two large African-American cohorts, including patients with LN-ESRD, suggested that increased allelic frequency of *APOL1* G1/G2 alleles in African-Americans might be the main genetic factor responsible for the poor prognosis of these patients with LN.^6^ To date, only one genome-wide association study (GWAS) has directly focused on LN among female patients with SLE of European descent.^7^ Besides the genes connected to LN identified in this GWAS, other genes associated to SLE, such as *FCGR*, *STAT4* and *BANK1*, have been validated in independent patient cohorts demonstrating association with LN and LN severity.^8–11^ Despite these recent advances, genetic risk factors for LN and the progression to ESRD have not been fully delineated.

Herein, we explored novel genetic associations predisposing to LN subphenotypes and ESRD in SLE by targeting regulatory and coding regions of 1832 immunological pathway genes in a cohort of Swedish patients with SLE, where SLE patients without LN were used as the comparator group. We identified a novel genetic locus associated with SLE-ESRD, and replicated findings in patients from different ancestral groups using a multiethnic cohort. Functional annotation and epigenetic analyses provided insight into the regulatory potential of variants at this locus, suggesting its potential as a target for modulating genetic risk of SLE-ESRD.

## Methods

### Participants

The discovery study population comprised 1167 patients with SLE, recruited at the Rheumatology clinics at four university hospitals in Sweden. Detailed characteristics of Swedish patients with SLE are reported in Yavuz *et al*.^12^ The quality controlled discovery dataset used in subsequent analyses comprised 958 patients with SLE including 338 (35.2%) with LN.^13^ All subjects provided consent to participate in the study. The replication cohort included 1244 SLE patients from a University of California, San Francisco (UCSF) multiethnic study,^9^ which included samples from the GENLES study.^14^ The multiethnic replication cohort included East Asian, Hispanic, North European, South European and African-American patients from established lupus cohorts from the USA, Australia, Spain and Mexico. A total of 1226 patients with SLE including 603 with LN had complete phenotypic data and were available for analyses of the multiethnic replication cohort.

All patients in both cohorts fulfilled the 1997 American College of Rheumatology (ACR) classification criteria for SLE.^15^ LN was defined by the ACR renal criterion. We stratified patients with LN into three subphenotypes (proliferative, pure membranous and ESRD) with the assumption of increasing power for specific risk loci. Proliferative and membranous LN definitions were based on the classification of the biopsies according to the 1982 WHO/International Society of Nephrology/Renal Pathology Society (ISN/RPS 2003) classes.^16 17^ Proliferative LN was defined as WHO Class III/IV and pure membranous by WHO Class V. ESRD was defined as patients who required renal replacement therapy, dialysis or transplantation. The non-LN group of patients with SLE was defined as patients not fulfilling the ACR renal criterion.

### Genetic analyses

In the discovery cohort, DNA was extracted from blood samples of all study participants, and target capturing for sequencing was performed using a NimbleGen array, including coding and regulatory regions of 1832 genes selected based on their involvement in immunological pathways. The design and the implementation of this capturing array, as well as subsequent sequencing experiments and quality control (QC), have been outlined elsewhere.^13^ After stringent QC filtering, a total of 97 376 single nucleotide variants (SNVs) with a minor allele frequency (MAF) >0.01 and 958 patients with SLE remained for the analyses of the discovery cohort. For this cohort, we generated principal components (PCs) for population stratification evaluation using EIGENSOFT as previously described, where the three most significant PCs were found to explain most of the population variation.^13 18^

Imputation of additional variants in the discovery cohort was performed employing the Sanger imputation service with the Haplotype reference consortium r1.1 reference panel described in McCarthy *et al*^19^ and the ‘pre-phase with EAGLE2 and impute’ pipeline^20^ after applying a 0.99 SNV call rate filter. Imputed genotype calls with a genotype probability score below 0.9 were set to missing and only variants with (1) info score ≥0.8, (2) MAF >0.01, (3) no significant deviation from Hardy-Weinberg equilibrium (HWE; p>0.0001) and (4) call rate >99% were retained. Genotypes were subsequently coded as hard called genotypes. After applying of these quality control parameters, 245 235 SNVs, with MAF >0.01 remained in the discovery dataset for the association analyses (97 376 directly genotyped and 147 859 imputed SNVs). The multiethnic replication cohort had previously been genotyped using the Affymetrix LAT1 World array, as detailed in Lanata *et al*.^9^

### Single variant association analyses, meta-analysis and power calculations

In the discovery cohort, single variant association analysis for each LN phenotype (SLE patients with LN, proliferative LN, membranous LN or SLE-ESRD vs SLE patients without LN) was performed using a logistic regression model in PLINK V.1.07,^21^ with three population structure PCs, sex and age at diagnosis as covariates. Due to the exploratory nature of the study, all SNVs with a suggestive p value ≤1×10^−4^ (n=155) in the analyses comparing proliferative LN, membranous LN and SLE-ESRD to SLE patients without LN in the discovery cohort were selected for analysis in the replication cohort. We also included variants located up to 100 kb upstream and downstream of the signals. This resulted in 36 candidate genomic regions for further evaluation. Permutation testing, to generate empirical logistic regression p values, was performed in PLINK using label swapping and the default adaptive permutation approach.

To increase the set of overlapping variants between the discovery and replication cohorts, the replication cohort genotype data were imputed and quality controlled using the same method as described above for the discovery cohort, applying the following thresholds: genotype probability >0.9, info score >0.8, MAF >0.01, HWE p>0.0001 and call rate >0.95. After imputation and QC, the replication dataset for the 36 candidate regions detected in the discovery cohort contained 45 083 SNVs. Single variant association analysis for each LN phenotype in the replication cohort was performed in PLINK using logistic regressions with three PCs for population stratification, sex and age at diagnosis as covariates. Also for the replication study, permutation testing was performed to assess the association p values. Additionally, logistic regression analyses were performed separately for each ethnicity (Asian, black, Hispanic, European). Meta-analysis was carried out for variants in the 36 candidate regions that were overlapping between the discovery and replication datasets. Results from each ethnicity were considered separately, and for the meta-analysis, we applied a random effect model using DerSimonian-Laird estimators and Wald-type tests and CIs using the R package metaphor.^22^

Statistical power for genetic associations in the discovery and replication studies for the three LN phenotypes was calculated using the genpwr R package^23^ using logistic regression assuming an additive model with alpha_discovery_=0.0001, alpha_replication_=0.05, MAF=0.05–0.25, OR=1.5–5. Results indicated that genetic associations for common SNVs of moderate effects could be well detected in both the discovery and replication studies for proliferative LN, whereas large effect sizes would be required for detection of membranous LN and SLE-ESRD associations (online supplemental figure S1, S2).

10.1136/lupus-2022-000752.supp1 Supplementary data

**Figure 1:**
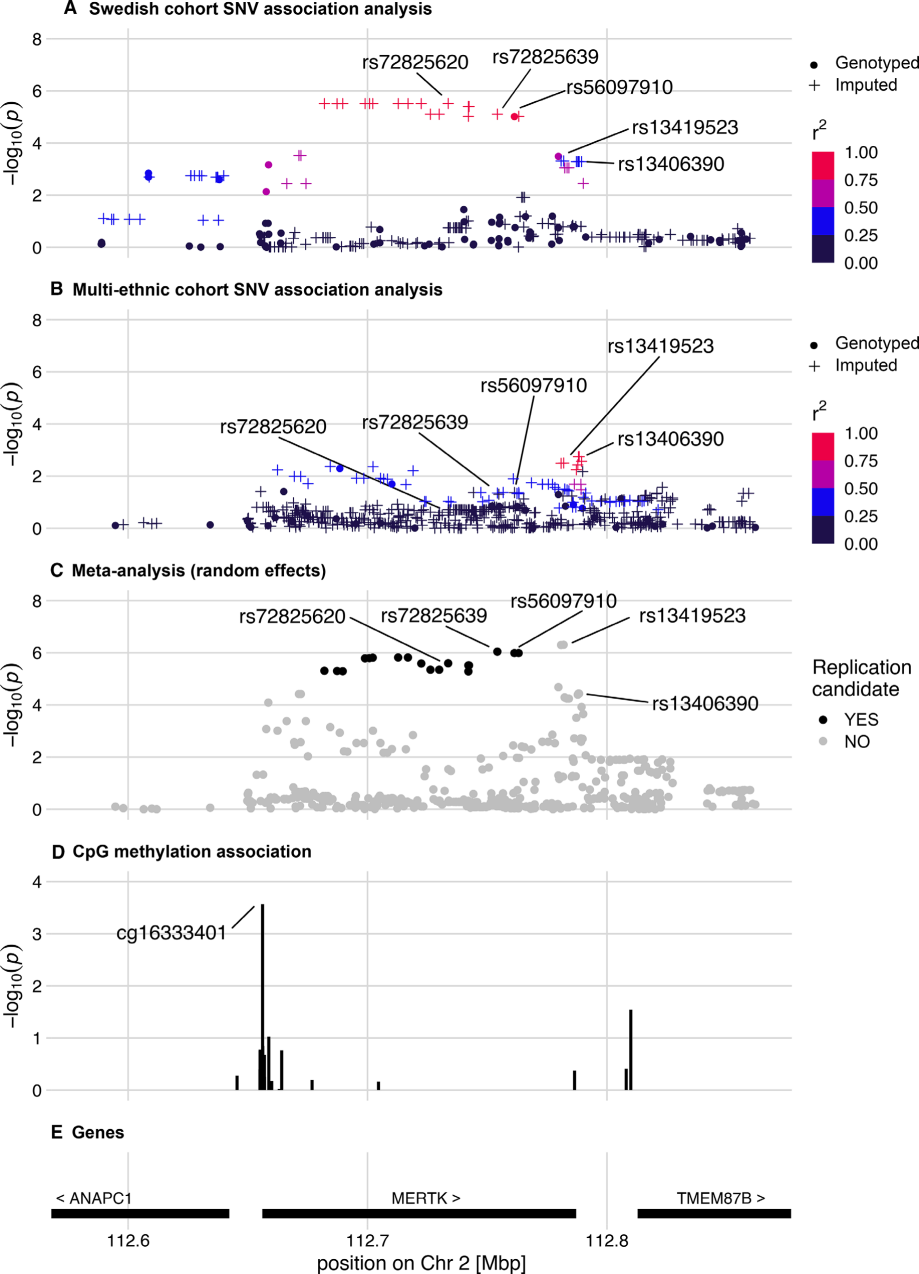
Regional association plots of the *MERTK* region. The regional association plots display results of the analysis of SLE-ESRD (n=35;73) versus SLE without nephritis (n=620;623) in (A) discovery and (B) replication datasets, respectively. Imputed SNVs are indicated by plus signs. (C) Meta-analysis of discovery and replication SLE-ESRD association analyses. All overlapping SNVs analysed in the discovery and replication cohorts are included. For the meta-analysis candidate, SNVs from the discovery cohort analysis are indicated in black. (D) Epigenetic analysis in SLE-ESRD (n=20) versus non-renal SLE (n=302) revealed a CpG site with decreased methylation in ESRD. (E) Genes and their chromosomal position in the region. ESRD, end-stage renal disease; SNV, single nucleotide variant

### Analysis of DNA methylation

Peripheral blood DNA methylation was interrogated using the Illumina HM450k array. Data acquisition, preprocessing, QC, normalisation of methylation data and estimation of relative blood cell type distribution have been described previously.^24^ Differential DNA methylation for CpG sites at the loci of interest between SLE-ESRD (n=20) and non-renal SLE (n=302) was tested using a linear regression model including age at sampling, sex, blood cell type distribution and HM450k BeadChip as covariates, with significance defined at p<0.0028 after Bonferroni correction for multiple testing (0.05/18 tests).

### Functional annotation

To evaluate the potential functional impact of the identified associated variants, we performed detailed functional annotation by using a combination of in silico tools and public datasets. HaploReg v4.1 was used to perform epigenomic annotation.^25^ To explore expression quantitative trait loci (eQTL) across different human tissues, we used several public databases: Genotype-Tissue Expression (GTEx) project,^26^ Blood NESDA NTR Conditional eQTL Catalog,^27^ Blood eQTL^28^ and RegulomeDB.^29^ In addition, GTEx data were also queried to identify genetic variants regulating DNA splicing (splicing QTL, sQLT). The transcription factor binding predictions were analysed using the sTRAP online tool with default parameters.^30^ Genomic overlap between differentially methylated CpG sites with chromatin marks and DNase hypersensitivity sites was analysed using the NIH Roadmap Epigenomics Programme/ENCODE database.^31^ Using the online tool Capture Hi-C plotter (CHiCP), chromatin interactions between SNVs and gene promoter regions were evaluated.^32^

## Results

Clinical characteristics of the 2184 patients who fulfilled the 1997 ACR SLE criteria^15^ are summarised in table 1. Of the 941 patients with LN, 428 had a renal biopsy and were stratified into proliferative and membranous LN based on renal histopathology according to the WHO/ISN/RPS classification system.^16^ SLE-ESRD was defined as patients who required renal replacement therapy, dialysis or transplantation.

**Table 1:** Patients with SLE, characteristics in discovery and replication cohorts

	Discovery (Sweden)		Replication (multiethnic)			
	LN (n=338)	SLE non-LN (n=620)	P value†	LN (n=603)	SLE non-LN (n=623)	P value†
Females, n (%)	261 (77.2)	565 (91.1)	<0.01	533 (88.4)	583 (93.6)	<0.01
Age at diagnosis, year (SD)*	30.8 (15.4)	38.8 (15.7)	<0.01	28.2 (11.8)	34.9 (12.9)	<0.01
SLE disease duration, year (SD)*	17.1 (11.5)	16.0 (12.1)	0.19	9.6 (8.3)	7.8 (8.3)	0.18
Kidney biopsy (%)	257 (76.0)			171 (28.4)		
Proliferative (%)	153 (45.3)			93 (15.4)		
Pure membranous (%)	41 (12.1)			40 (6.6)		
End-stage renal disease (%)	35 (10.4)			73 (12.1)		
Hypertension (%)	144 (42.6)	151 (24.4)	<0.01			
Diabetes mellitus (%)	9 (2.7)	30 (4.8)	0.086			

*Mean, SD.

†Independent samples t-test. Data are presented as mean (SD) or n (%), unless otherwise indicated. Patients fulfilled at least 4 of 11 ACR criteria for SLE.^15^ LN was defined by the ACR renal criterion or renal biopsy. Proliferative LN was defined as WHO Class III/IV, and pure membranous by WHO Class V. ESRD was defined as patients who required renal replacement therapy, dialysis or transplantation. The non-LN group of patients with SLE was defined as patients not fulfilling the ACR renal criterion.

ACR, American College of Rheumatology; ESRD, end-stage renal disease; LN, lupus nephritis.

### Potentially novel associations with proliferative, membranous and end-stage lupus nephritis

We then performed a case-case analysis using these more homogeneous LN phenotypes proliferative LN, pure membranous LN and SLE-ESRD versus SLE without renal involvement (online supplemental table S1). The strongest associated directly genotyped variant within each locus was used as the index SNV of association. Thirty-seven SNVs showed suggestive evidence of associations with proliferative LN in the discovery cohort (p≤1×10^-4^, online supplemental table S1A). Associations were observed with proliferative LN and *OAS2* (2′-5′-oligoadenylate synthetase, rs1293765, p=1.8×10^-5^), *APOA1BP/NAXE* (apolipoprotein A-I binding protein, rs942960, p=2.8×10 ^-5^), *AK8* (adenylate kinase, rs192593197, p=3.5×10^-5^) and *PRDM1* (PR domain 1, rs6924535, p=5.1×10^-5^). *OAS2* and *PRDM1* have been implicated in the Toll-like receptor (TLR) signalling pathway and SLE, respectively.^33–35^ Interestingly, the most significant potentially novel SNVs within the *PRDM1* gene, which encodes B-lymphocyte-induced maturation protein 1 (BLIMP-1), are not in linkage disequilibrium (LD) with previously reported SLE-associated SNVs (rs548234 and rs6568431).

10.1136/lupus-2022-000752.supp2 Supplementary data

Among the 39 variants that exceeded the suggestive level of significance for pure membranous LN in the discovery cohort, the strongest association corresponded to an intronic variant located in *LTF* (lactotransferrin, rs6776245, p=1.6×10^-6^, online supplemental table S1B). *LTF* encodes a major iron-binding protein found in secondary granules of neutrophils. Another candidate gene is *MMS19* (MMS19 homologue, cytosolic iron-sulfur assembly component, rs116933945, p=8.4×10^-5^).

Finally, we observed suggestive associations with SLE-ESRD at 79 SNVs in the discovery cohort (p≤1×10^-4^, online supplemental table S1C). The strongest signals of association originated from several variants in complete LD located in the *MERTK* gene region (MER proto-oncogene, tyrosine kinase gene), which encodes tyrosine-protein kinase Mer (top SNV rs72825620, p=3.0×10^-6^); of which one was directly genotyped (index SNV: rs56097910, p=9.5×10^-6^) (figure 1A). We found that this *MERTK* variant, rs56097910, was significantly enriched among patients with SLE who developed ESRD (MAF=0.13) compared with patients without LN SLE (MAF=0.02) (online supplemental table S1C). The other loci with suggestive associations with SLE-ESRD have previously been related to type 1 diabetes-related ESRD (*AFF3*, AF4/FMR2 family member 3),^36^ type 2 diabetes (*THADA*, THADA armadillo repeat containing)^37^ and potassium channels (*GRK5*, G protein-coupled receptor kinase 5)^38^ (online supplemental table S1C).

### Multiethnic replication cohort and meta-analysis

To validate our findings, all regions containing SNVs with an association p value of <1×10^-4^ for each of the three LN traits in the discovery cohort were selected and examined in a multiethnic replication cohort. Genotype imputation was performed in both cohorts to increase the set of overlapping variants between the discovery and replication cohorts. We first performed a logistic regression analysis in the full replication cohort, followed by logistic regression analyses in each ethnicity independently (online supplemental table S1). Lastly, we performed a meta-analysis with a random effects model using the results of both discovery and replication cohorts (online supplemental table S1). Additionally, permutation testing was performed in both the discovery and replication cohorts to asses the p values, which remained largely the same (online supplemental table S1).

In proliferative LN, the strongest signal within *PRDM1* (rs6924535, p_multiethnic_=0.029) and two SNVs in complete LD (rs942960, rs942961; p_multiethnic_=0.037) located in *APOA1BP* (*NAXE*) were nominally significant in the multiethnic replication cohort (table 2, online supplemental table S1A). In the meta-analysis, the strongest association for directly genotyped variants in *PRDM1* came from three polymorphisms in high LD (r^2^=0.92, index SNV: rs6924535, p_meta_=1.6×10^-5^, OR=0.58; table 2). The two aforementioned SNVs located in *APOA1BP* (*NAXE*) were also significant in the meta-analysis (p_meta_=1.2×10^-5^, OR=2.64). For membranous LN, none of the SNVs identified in the discovery cohort replicated in the multiethnic cohort. However, the biopsy rate was lower in the replication cohort, hampering replication of the associations for proliferative and membranous LN (76% vs 28%, discovery and replication cohorts, respectively, table 1).

**Table 2:** Replication cohort and meta-analysis results for replicated loci for proliferative lupus nephritis and end-stage renal disease in SLE

		Discovery cohort (Sweden)				Replication cohort (multiethnic)**				Meta-analysis††		
Locus	SNV	MAF	MAF	P value	OR (95% CI)	MAF	MAF	P value	OR (95% CI)	P value	OR	I^2^ %
*Proliferative LN vs SLE-non-LN*		pLN+ n=153	LN- n=620			pLN+ n=93	LN- n=623					
*APOA1BP*	rs942960	0.08	0.03	2.76E-05	3.23 (1.87 to 5.59)	0.08	0.05	0.037	1.99 (1.04 to 3.8)	1.16E-05	2.64	0
*APOA1BP*	rs942961	0.08	0.03	2.76E-05	3.23 (1.87 to 5.59)	0.08	0.05	0.037	1.99 (1.04 to 3.8)	1.16E-05	2.64	0
*PRDM1*	rs1984224	0.25	0.38	6.14E-05	0.54 (0.4 to 0.73)	0.25	0.31	0.036	0.66 (0.44 to 0.97)	2.50E-05	0.58	0
*PRDM1*	rs6924535	0.26	0.38	5.08E-05	0.53 (0.39 to 0.72)	0.25	0.30	0.029	0.65 (0.44 to 0.96)	1.58E-05	0.59	0
*PRDM1*	rs535780	0.26	0.38	7.88E-05	0.54 (0.4 to 0.73)	0.30	0.34	0.019	0.63 (0.43 to 0.93)	3.19E-03	0.59	30

*ESRD vs SLE non-LN*		ESRD+ n=35	LN- n=620			ESRD+ n=73	LN- n=623					
*MERTK*	rs72825639	0.13	0.02	7.84E-06	7.58 (3.12 to 18.43)	0.05	0.03	0.044	2.57 (1.02 to 6.46)	8.95E-07	5.50	0
*MERTK*	rs56097910	0.13	0.02	9.52E-06	7.32 (3.03 to 17.68)	0.05	0.03	0.043	2.59 (1.03 to 6.5)	1.03E-06	5.40	0
*MERTK*	rs72825650	0.13	0.02	9.49E-06	7.33 (3.03 to 17.68)	0.05	0.03	0.043	2.59 (1.03 to 6.5)	1.03E-06	5.40	0

*Multiethnic cohort, all ethnicities combined.

†Each replication population analysed separately by logistic regression, then included in a meta-analysis with the discovery cohort.

ESRD, end-stage renal disease; I^2^, I^2^-statistics for heterogeneity of studies; LN, lupus nephritis; MAF, minor allele frequency; pLN, proliferative LN.

We then focused our continued analyses on the outcome SLE-ESRD, as uniform data for this phenotype were available for both cohorts. In ESRD, the top directly genotyped SNV rs56097910 identified within *MERTK* in the discovery cohort had been imputed in the replication cohort. This SNV was also associated with ESRD in the replication cohort (p_multiethnic_=0.043; OR: 2.59; table 2, online supplemental table S1C), as were a number of additional variants (figure 1B, online supplemental table S2). In the meta-analysis, an imputed SNV (rs72825639) in complete LD with rs56097910 showed the strongest effect (p_meta_=8.9×10^-7^, OR=5.49) followed by rs56097910 (p_meta_=1.0×10^-6^, OR=5.40) (figure 1C, online supplemental table S2). Of note, this region also harboured an additional SNV rs13419523 (p_meta_=4.9×10^-7^, OR=3.90) which had not passed the discovery suggestive significance threshold (figure 1C, online supplemental table S2).

### Functional annotations and epigenetic enrichment analysis

Functional annotations of variants at the three replicated loci associated with proliferative LN and ESRD are shown in table 3. We focused the functional follow-up on the locus discovered for the outcome SLE-ESRD. The ESRD-associated genetic variants in *MERTK* reside in a non-coding region, suggesting that they might exert their effect on the disease through gene regulation. We therefore investigated DNA methylation at the *MERTK* locus in ESRD compared with non-renal SLE, and identified a CpG site cg16333401 with decreased methylation in ESRD (p=0.00027) (online supplemental table S3). This CpG site is located in close proximity to the transcription start site of *MERTK* (figure 1D–E). We also analysed genomic overlap of differential methylation with six different histone marks (H3K4me1, H3K4me3, H3K36me3, H3K9me3, H3K27ac and H3K27me3) and DNAse hypersensitivity sites in reference lymphocytes using ENCODE (online supplemental table S3). We observed that in both T and B lymphocytes, the differentially methylated sites overlapped with histone marks of active enhancers (H3K4me1 and H3K4me3) and with a DNAse hypersensitivity region, suggesting a potentially functional role in transcriptional regulation.

**Table 3:** Functional annotations of top SNVs at the three loci associated with lupus nephritis subphenotypes

Locus	SNV	RegulomeDB*† score	Promoter histone marks	Enhancer histone marks	DNAse hypersensitivity	TFs bound‡	eQTL in blood cells	eQTL in other tissues
*PRDM1*	rs6924535	0.703	Yes	Yes	Yes			
*APOA1BP*	rs942960	0.554		Yes	Yes		*APOA1BP*	*GPATCH4*
*MERTK*	rs56097910	0.579	Yes	Yes	Yes	EBF1, PAX5C20	*MERTK*	*MERTK, TMEM87B*
*MERTK*	rs13419523	0.705		Yes	Yes	MAFK	*MERTK*	*MERTK, FBLN7*

*The RegulomeDB probability score is ranging from 0 to 1, with 1 being most likely to be a regulatory variant.

†Boyle *et al.*^29^

‡Transcription factors (TFs) bound in ChIP-Seq experiments (ENCODE Project Consortium, 2011).

eQTL, expression quantitative trait locus; SNV, single nucleotide variant.

Given that epigenetic marks may correlate with gene expression changes, we searched through several public databases of eQTLs (detailed in the Methods section) to explore the relationship between the top SNVs rs56097910 and rs13419523, and *MERTK* expression. Besides renal cortex (online supplemental figure S3), we found association with expression change of *MERTK* in whole blood (online supplemental figure S3, S4), fibroblasts (p=1.4×10^-7^) and in other tissues such as lung (p=2.9×10^-27^), and subcutaneous adipose tissue (p=1.4×10^-36^). The role of the region with the ESRD-associated top variants (rs56097910, rs13419523) in *MERTK* regulation is also supported by their physical interactions with the *MERTK* promoter in multiple cells such as macrophages, monocytes, B and T cells (online supplemental figure S3, S4). Next, we determined which transcription factors (TFs) have binding sites that might be affected by rs56097910 or rs13419523. We identified differences in binding of several TFs such as zinc finger 423 (ZNF423/ROAZ) and SMAD4 which are involved in TGF-β signalling and the Th1 differentiation pathway (online supplemental table S4).

## Discussion

Herein, we report results of a large genetic association study that aims to identify novel genetic variants contributing to the risk of developing LN subphenotypes and ESRD among patients with SLE. We identified a novel genetic region, *MERTK*, associated with SLE-ESRD. Moreover, our results replicate and extend across ethnicities. To the best of our knowledge, this is the first report of the association of *MERTK* as a susceptibility locus for ESRD in SLE patients with LN, in at least two different ancestries.

Renal damage including ESRD is one of the major predictors of mortality in SLE.^39 40^ Although molecular mechanisms for different histopathologies in LN have not been fully elucidated, the identification of the genetic risk factors for the subgroup of patients with LN who developed ESRD may lead to a better risk assessment and future targeting of the relevant pathways resulting in improved survival. We found that a variant, rs56097910, in *MERTK* is significantly enriched among patients with SLE who developed ESRD compared with patients without LN SLE in our discovery cohort. When we sought to replicate this variant in an independent multiethnic SLE cohort from the USA, we observed an increased MAF in ESRD compared with non-renal SLE across populations, except for those with African ancestry.

In addition to rs56097910, we found several variants within *MERTK* that are associated with ESRD (figure 1), further strengthening a role for this region in ESRD. MERTK is a member of the Tyro3/Axl/Mer (TAM) receptor kinase family and the main apoptotic cell receptor on macrophages.^41 42^ MERTK has been implicated in the regulation of innate immune response through efferocytosis, and is linked to changes in cytokine production, including interleukin-10 (IL-10), transforming growth factor-β (TGF-β), IL-6 and IL-12.^43 44^ Furthermore, it has been shown to play an important role in inhibition of TLRs-mediated innate immune response by activating STAT1, which contributes to the inflammatory negative feedback signals by inducing the production of suppressors of cytokine signalling SOCS1 and SOCS3.^45^

Our functional annotation analyses strongly suggested a regulatory role of rs56097910 and/or other significant variants in *MERTK*. The index SNV, rs56097910, is an eQTL for *MERTK* expression in multiple tissues including kidney, where the minor allele is associated with increased gene expression. In addition, the finding of overlap with active chromatin epigenetic marks in this risk locus reflects a robust functional signature. It remains unclear how the increased *MERTK* expression relates to a severe SLE outcome such as ESRD. LN is characterised by recurring injury-repair cycles because of unresolved inflammation.^4^ One possible explanation might relate to the activation of STAT1, as STAT1 also acts as a transducer of multiple cytokines such as IFN (α/β/γ).^42 43^ Alternatively, MERTK-expressing macrophages may play a key role in dysregulated repair in kidneys. We hypothesise that MERTK suppresses inflammation via increased efferocytosis that may promote fibrosis—partly through TGF-β—a similar mechanism shown in tissue repair following liver injury and idiopathic pulmonary fibrosis in which MERTK-expressing macrophages aid the process.^46 47^ Of note, recent studies show that plasma soluble tyrosine-protein kinase Mer (sMer) levels correlate with the disease activity and severity in patients with LN.^48–51^ However, the relative contribution of *MERTK* gene variants to SLE-ESRD compared with other risk factors is difficult to estimate, but we noticed that the vast majority of patients with ESRD received treatment for hypertension, unlike in the SLE non-LN group where a minority of patients had such treatment (80% vs 24%, t-test p value <0.01). In contrast, there was no difference in prevalence of diabetes between these two groups. Therefore, further research that focuses on elucidating the role of this genetic association with *MERTK* and its effect on SLE-ESRD pathophysiology is warranted.

The results also revealed a new variant, rs6924535, in an intronic region of the *PRDM1* gene and its association with proliferative LN, which was replicated in the US multiethnic cohort. The *PRDM1* gene encodes a protein (BLIMP-1) that is an essential modulator of dendritic cell function and a repressor of the interferon β gene.^52^ Although BLIMP-1 drives B cells into antibody secreting cells,^53^ its expression in peripheral blood B cells appears to be low and is not affected by genetic variants.^54^ In addition, variants in the intergenic region between the *PRDM1* and *ATG5* genes are associated with risk for SLE.^55 56^ Of note, the protective allele of rs6924535 is not in LD with previously confirmed variants associated with an increased risk for SLE in this region. We also identified another new genetic signal within *APOA1BP* (*NAXE*) on chromosome 1q22, which also replicated in the US multiethnic cohort. The encoded product, apolipoprotein A1 binding protein, interacts with apolipoprotein A-1 and functions in cholesterol transport. The related pathway is involved in the protection of the cell from reactive oxygen species. The eQTL analysis of proliferative LN risk variants in this locus revealed higher expression of *NAXE* in blood monocytes and multiple tissues (data not shown). With regard to membranous LN-associated signals, none of the variants were replicated, likely due to the small sample size in each ethnicity.

The major strength of this study is the utilisation of two large SLE cohorts from different ancestries. It is worth mentioning that most patients with LN in our discovery cohort had a corresponding renal biopsy (76%), allowing for assessment of specific histopathological subtypes. Some limitations should also be acknowledged. We targeted more homogeneous LN phenotypes hypothesising that this would increase power for specific loci; however, our approach might result in limited statistical power for analysis in some subphenotypes, due to low biopsy rates in the replication cohort. Our experiment covered 1832 genes, it is still limited by virtue of targeting some of the genes and pathways that are known to be involved in inflammation. Finally, our SNV analysis did not incorporate all known risk loci for chronic kidney diseases.

In summary, we identified a novel SLE-ESRD susceptibility locus, containing the *MERTK* gene, in a large Swedish SLE cohort and corroborated our findings in a multiethnic SLE cohort. We characterised the functional potential of this gene using in silico tools. Our analyses highlighted immune cell types that are involved in mediating genetic risk in ESRD associated with LN. Of note, *MERTK* does not confer an increased risk for SLE per se. Given that ESRD is one of the main predictors of mortality in lupus and the ability to predict progression to ESRD eventually may yield therapeutic targets to prevent it, our results support MERTK as a promising target for preventing ESRD in patients with LN.

10.1136/lupus-2022-000752.supp3 Supplementary data

## Data Availability

Data are available upon reasonable request. The datasets generated during the current study are not publicly available due to them containing information that could compromise research participant privacy and consent, but are available from the corresponding authors on reasonable request and on a collaborative basis.
