# Supplementary material for: Mer-tyrosine kinase: a novel susceptibility gene for SLE related end-stage renal disease
Source: Lupus Sci Med. 2022 Nov 4;9(1):e000752. doi: 10.1136/lupus-2022-000752 (PMC9639142; doi:10.1136/lupus-2022-000752)
Supplement: Supplementary data [file lupus-2022-000752supp001.pdf]

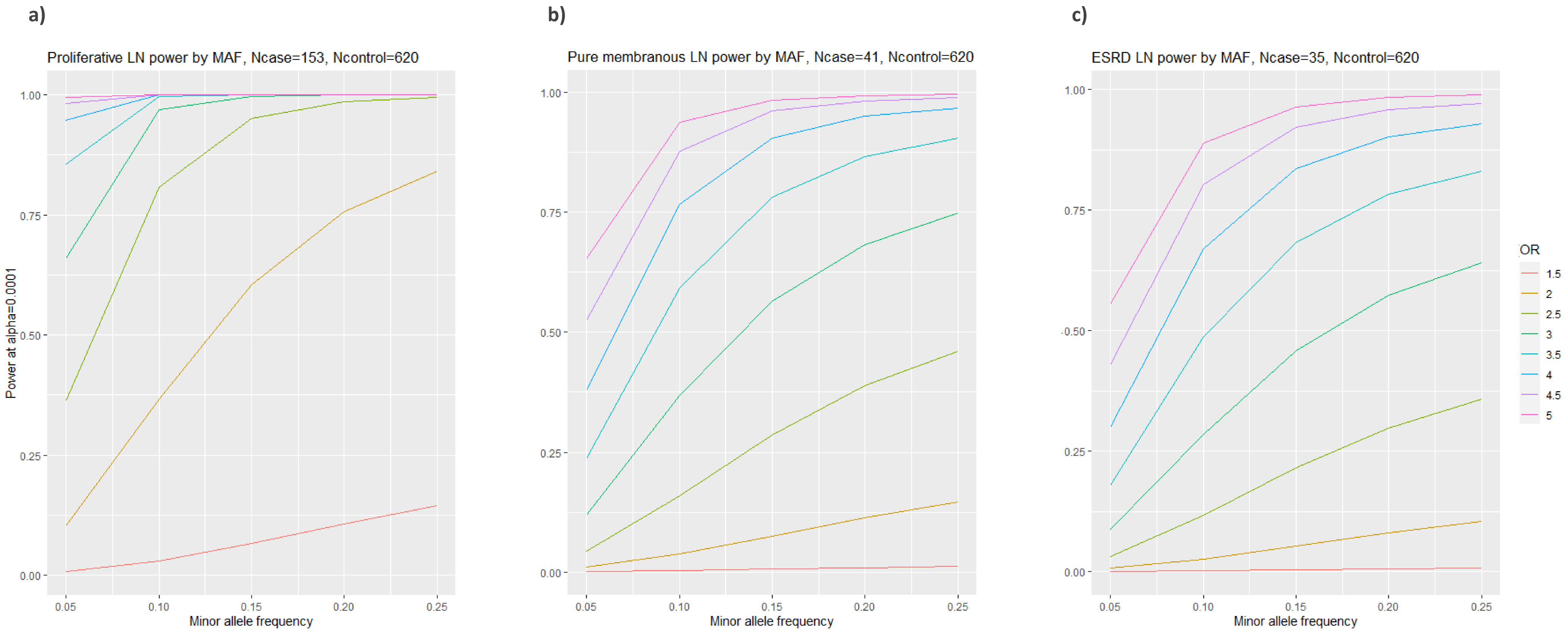

Figure S1. Statistical power, discovery study. Results of power calculations for genetic association assuming an additive model, alpha=0.0001, using logistic regression for a) Proliferative LN vs. non-LN SLE. b) Pure membranous LN vs. non-LN SLE. c) End-stage renal disease vs. non-LN SLE. LN: lupus nephritis; MAF: minor allele frequency; OR: odds ratio.

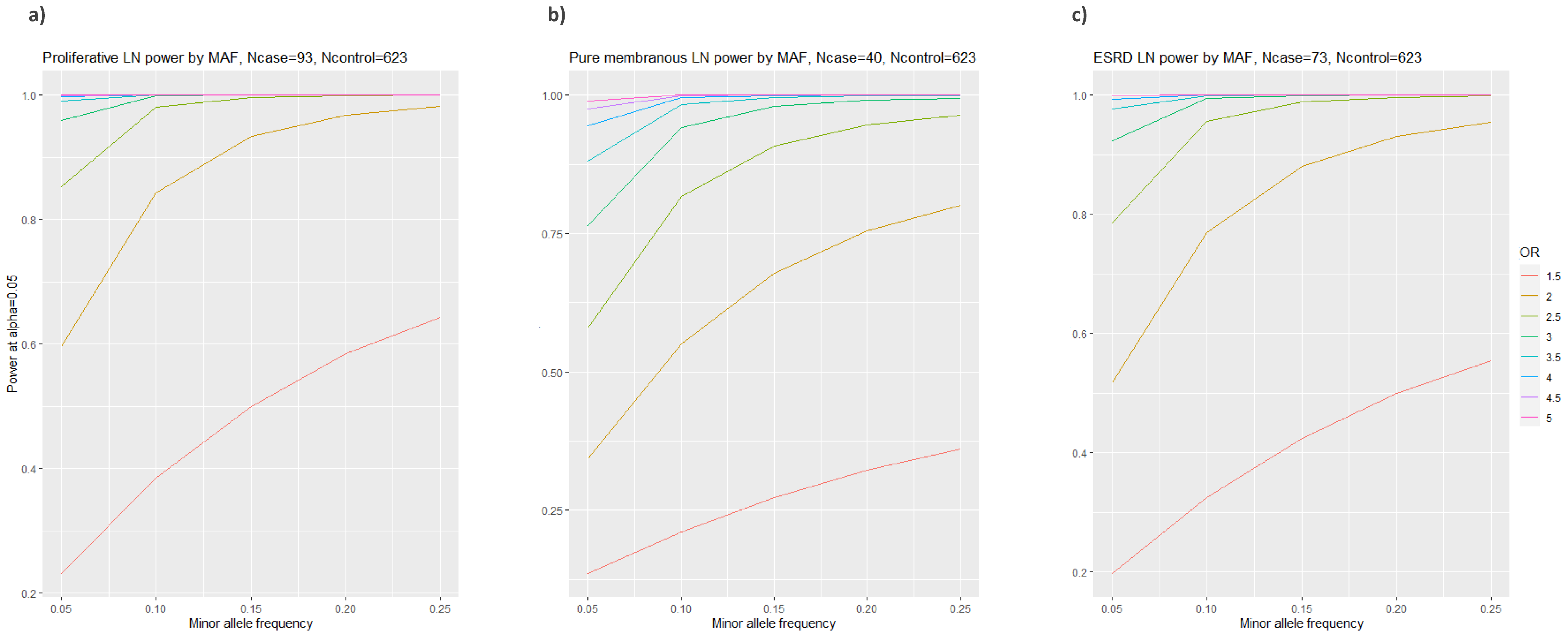

Figure S2. Statistical power, replication study. Results of power calculations for genetic association assuming an additive model, alpha=0.05, using logistic regression for a) Proliferative LN vs. non-LN SLE. b) Pure membranous LN vs. non-LN SLE. c) End-stage renal disease vs. non-LN SLE. LN: lupus nephritis; MAF: minor allele frequency; OR: odds ratio.

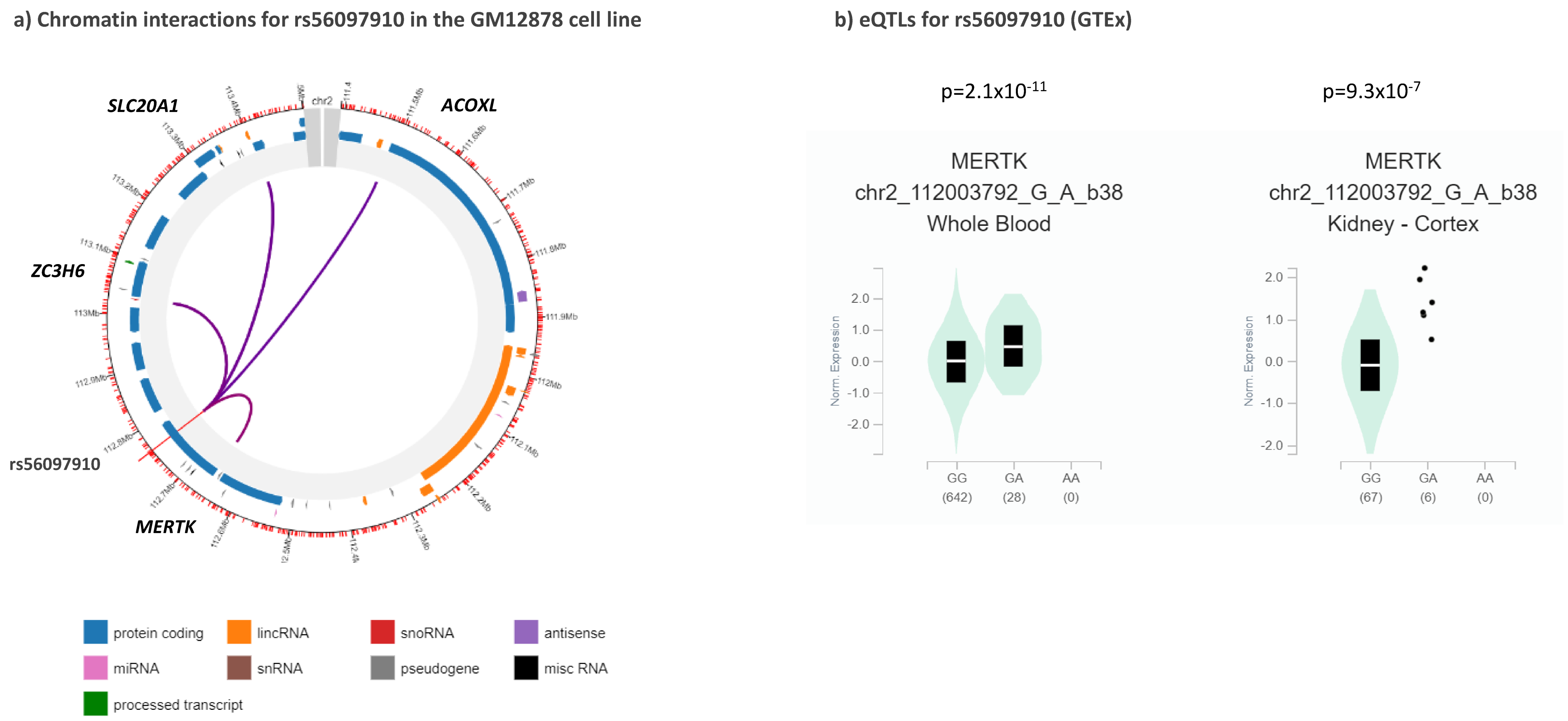

Figure S3. Functional annotation results of *MERTK* rs56097910. a) Circular view of chromatin interaction between rs56097910 and *MERTK* obtained from Capture Hi-C data (https://www.chicp.org/). The outer circle contains a genomic scale and restriction enzyme (Hind III) cleavage sites, inside of which gene type with colour depicting biotype are represented. This interaction is observed in GM128787 lymphoblastoid cell line. The red line indicates a strong confidence for this interaction. b) Violin plot representing the difference in expression levels of *MERTK* depending on rs56097910 genotypes obtained from the GTEx project.

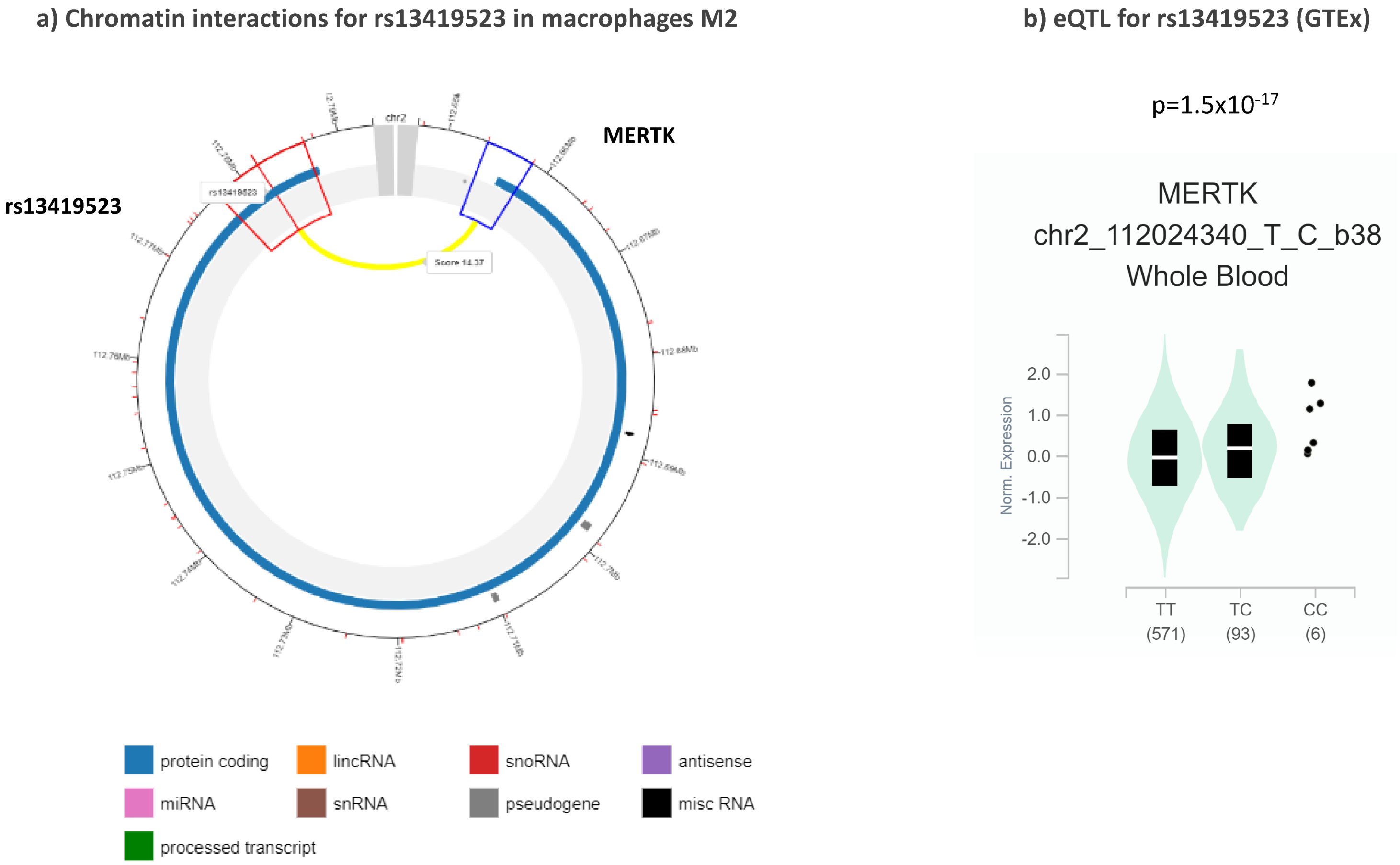

Figure S4. Functional annotation results of *MERTK* rs13419523. a) Circular view of chromatin interaction between rs13419523 and *MERTK* obtained from Capture Hi-C data (https://www.chicp.org/). The outer circle contains a genomic scale and restriction enzyme (Hind III) cleavage sites, inside of which gene type with colour depicting biotype are represented. This interaction is observed in macrophages M2 cell line. The red line indicates a strong confidence for this interaction. b) Violin plot representing the difference in expression levels of *MERTK* depending on rs13419523 genotypes obtained from the GTEx project.
