# Supplementary material for: Mer-tyrosine kinase: a novel susceptibility gene for SLE related end-stage renal disease
Source: Lupus Sci Med. 2022 Nov 4;9(1):e000752. doi: 10.1136/lupus-2022-000752 (PMC9639142; doi:10.1136/lupus-2022-000752)
Supplement: Supplementary data [file lupus-2022-000752supp003.pdf]

Collaborators

The DISSECT consortium: Sule Yavuz (Department of Medical Sciences, Rheumatology, Uppsala University, Uppsala, Sweden), Pascal Pucholt (Department of Medical Sciences, Rheumatology, Uppsala University, Sweden), Johanna K. Sandling (Department of Medical Sciences, Rheumatology, Uppsala University, Uppsala, Sweden), Fabiana H.G. Farias (Science for Life Laboratory, Department of Medical Biochemistry and Microbiology, Uppsala University, Uppsala, Sweden, and Department of Psychiatry, Washington University, St. Louis, MO, USA), Sergey V. Kozyrev (Science for Life Laboratory, Department of Medical Biochemistry and Microbiology, Uppsala University, Uppsala, Sweden), Maija-Leena Eloranta (Department of Medical Sciences, Rheumatology, Uppsala University, Uppsala, Sweden), Matteo Bianchi (Science for Life Laboratory, Department of Medical Biochemistry and Microbiology, Uppsala University, Uppsala, Sweden), Leonid Padyukov (Division of Rheumatology, Department of Medicine, Karolinska Institutet and Karolinska University Hospital, Stockholm, Sweden), Ann-Christine Syvänen (Department of Medical Sciences, Molecular Medicine and Science for Life Laboratory, Uppsala University, Uppsala, Sweden), Andreas Jönsen (Lund University, Skane University Hospital, Department of Clinical Sciences Lund, Rheumatology, Lund, Sweden), Iva Gunnarsson (Division of Rheumatology, Department of Medicine Solna, Karolinska Institutet, Karolinska University Hospital, Stockholm, Sweden), Elisabet Svenungsson (Division of Rheumatology, Department of Medicine Solna, Karolinska Institutet, Karolinska University Hospital, Stockholm, Sweden), Solbritt Rantapää-Dahlqvist (Department of Public Health and Clinical Medicine/Rheumatology, Umeå University, Umeå, Sweden), Anders A. Bengtsson (Lund University, Skane University Hospital, Department of Clinical Sciences Lund, Rheumatology, Lund, Sweden), Christopher Sjöwall (Department of Biomedical and Clinical Sciences, Division of Inflammation and Infection, Linköping University, Linköping, Sweden), Helena Enocsson (Department of Biomedical and Clinical Sciences, Division of Inflammation and Infection, Linköping University, Linköping, Sweden), Dag Leonard (Department of Medical Sciences, Rheumatology, Uppsala University, Uppsala, Sweden), Kerstin Lindblad-Toh (Science for Life Laboratory, Department of Medical Biochemistry and Microbiology, Uppsala University, Uppsala, Sweden and Broad Institute of MIT and Harvard, Cambridge, MA, USA), Lars Rönnblom (Department of Medical Sciences, Rheumatology, Uppsala University, Uppsala, Sweden), Johanna Dahlqvist (Science for Life Laboratory, Department of Medical Sciences and Department of Medical Biochemistry and Microbiology, Uppsala University, Uppsala, Sweden), Daniel Eriksson (Department of Medicine (Solna), Karolinska Institutet, and Department of Endocrinology, Metabolism and Diabetes Karolinska University Hospital, Stockholm, Sweden), Ingrid E. Lundberg (Division of Rheumatology, Department of Medicine and Center for Molecular Medicine, Karolinska Institutet, Stockholm, Sweden), Argyri Mathioudaki (Science for Life Laboratory,

Department of Medical Biochemistry and Microbiology, Uppsala University, Uppsala, Sweden), Jennifer Meadows (Science for Life Laboratory, Department of Medical Biochemistry and Microbiology, Uppsala University, Uppsala, Sweden), Jessika Nordin (Science for Life Laboratory, Department of Medical Biochemistry and Microbiology, Uppsala University, Uppsala, Sweden), Gunnel Nordmark (Department of Medical Sciences, Rheumatology, Uppsala University, Uppsala, Sweden), Marie Wahren-Herlenius (Department of Medicine, Division of Rheumatology, Karolinska Institutet, Karolinska University Hospital, Stockholm, Sweden and Broegelmann Research Laboratory, Department of Clinical Science, University of Bergen, Norway).

The ImmunoArray development consortium: Kerstin Lindblad-Toh (Science for Life Laboratory, Department of Medical Biochemistry and Microbiology, Uppsala University, Uppsala, Sweden and Broad Institute of MIT and Harvard, Cambridge, MA, USA), Gerli Rosengren Pielberg (Science for Life Laboratory, Department of Medical Biochemistry and Microbiology, Uppsala University, Uppsala, Sweden), Anna Lobell (Office for Medicine and Pharmacy, Uppsala University, Uppsala, Sweden), Åsa Karlsson (Science for Life Laboratory, Department of Medical Biochemistry and Microbiology, Uppsala University, Uppsala, Sweden), Eva Murén (Science for Life Laboratory, Department of Medical Biochemistry and Microbiology, Uppsala University, Uppsala, Sweden), Göran Andersson (Department of Animal Breeding and Genetics, Swedish University of Agricultural Sciences, Uppsala, Sweden), Kerstin M. Ahlgren (Department of Surgical Sciences, Uppsala University, Uppsala, Sweden), Lars Rönnblom (Department of Medical Sciences, Rheumatology, Uppsala University, Uppsala, Sweden), Maija-Leena Eloranta (Department of Medical Sciences, Rheumatology, Uppsala University, Uppsala, Sweden), Nils Landegren (Department of Medicine (Solna), Center for Molecular Medicine, Karolinska Institutet, Stockholm, Sweden and Science for Life Laboratory, Department of Medical Sciences, Uppsala University, Uppsala, Sweden), Olle Kämpe (Department of Medicine (Solna), Center for Molecular Medicine, Karolinska Institutet, Stockholm, Sweden, Department of Endocrinology, Metabolism and Diabetes Karolinska University Hospital, Stockholm, Sweden, Science for Life Laboratory, Department of Medical Sciences, Uppsala University, Uppsala, Sweden and KG Jebsen Center for autoimmune diseases, University of Bergen, Norway), Peter Söderkvist (Division of Cell Biology, Department of Biomedical and Clinical Sciences, Linköping University, Linköping, Sweden).

The multi-ethnic replication SLE cohort: Betty P. Tsao (Department of Medicine, Medical University of South Carolina, South Carolina, United States of America), Eric F Morand (School of Clinical Sciences, Monash University Faculty of Medicine, Nursing& Health Sciences, Melbourne, Australia), Kimberly E Taylor (Russell/Engleman Rheumatology Research Center, Department of Medicine, University of California San Francisco, San Francisco, USA), Marta E. Alarcon-Riquelme (GENYO.

Pfizer- University of Granada-Andalusian Government Center for Genomics and Oncological Research, PTS, Granada, Spain), Sharon A Chung (Russell/Engleman Rheumatology Research Center, Department of Medicine, University of California San Francisco, San Francisco, USA).
